# The Role of Offspring Genotype-by-Sex Interactions, Independently of Environmental Cues, on the Phenotype Traits of an Obese Swine Model

**DOI:** 10.3390/biology9120445

**Published:** 2020-12-04

**Authors:** Ana Heras-Molina, José Luis Pesantez, Susana Astiz, Consolación Garcia-Contreras, Marta Vazquez-Gomez, Beatriz Isabel, Cristina Ovilo, Antonio Gonzalez-Bulnes

**Affiliations:** 1 Subdirección General de Investigacion y Tecnologia, Instituto Nacional de Investigación y Tecnología Agraria y Alimentaria, Ctra, De La Coruña Km 7.5, 28040 Madrid, Spain; delasheras.ana@inia.es (A.H.-M.); jose.pesantez@ucuenca.edu.ec (J.L.P.); astiz.susana@inia.es (S.A.); congarcon@gmail.com (C.G.-C.); ovilo@inia.es (C.O.); 2 School of Veterinary Medicine and Zootechnics, Faculty of Agricultural Sciences, University of Cuenca, Avda. Doce de Octubre, Cuenca 010220, Ecuador; 3 Faculty of Veterinary Medicine, UCM, Ciudad Universitaria s/n, 28040 Madrid, Spain; martavazgomez@gmail.com (M.V.-G.); bisabelr@vet.ucm.es (B.I.)

**Keywords:** DOHaD, genotype, obesity, swine-model

## Abstract

**Simple Summary:**

The present study, comparing the postnatal development of purebred Iberian and crossbreds Iberian × Large White littermates born from purebred Iberian sows, allows us to discern phenotype traits driven by the genotype from features imposed by pre- and postnatal environment. The results obtained in this study support the well-known relevance of genotype but also evidence a paramount role of the interaction sex-by-genotype, with differential effects depending on the offspring genotype and sex.

**Abstract:**

The present study aimed to assess the importance of offspring genotype on postnatal development, independently of confounding factors related to prenatal environment and postnatal lifestyle, using a translational model of obesity and metabolic syndrome (the Iberian pig). Hence, we compared two genotypes (purebred Iberian and crossbreds Iberian × Large White), produced in one single maternal environment (pure Iberian mothers) through artificial insemination of Iberian sows with Iberian and Large White heterospermic semen and maintained in the same conditions during postnatal development. The results indicate that, under same pre- and postnatal environments, the interaction genotype-by-sex has a determinant role on offspring phenotype (i.e., growth and development, metabolic and antioxidant status and fatty acid composition of different tissues). These results may set the basis for future preclinical and clinical research on the differences in the metabolic phenotype among genotypes.

## 1. Introduction

Obesity was declared a global pandemic by the World Health Organization (WHO) in 2005, when the affected population reached 400 million of adults and at least 2.6 million of people were dying each year as a result of being overweight or obese (https://www.who.int/health-topics/obesity#tab=tab_1). Two main traits of obesity must be considered for the study and control of the disease and its consequences. First, morbidity and mortality linked to obesity are related to the appearance of non-communicable associated conditions (metabolic syndrome, diabetes and cardiovascular and renal diseases [1,2,3]) and to the worsening of the prognosis of communicable diseases, as recently evidenced for COVID-19 [4,5]. A second worrying aspect of the pandemic is the dramatic demographic change observed in the last years. The condition was traditionally described in adult individuals of wealthy populations from high-income countries. However, currently, the global changes in lifestyle and dietary patterns have modified its distribution and therefore obesity and associated disorders affect both children and adults of different socioeconomic classes in both developed and developing countries [6]. In fact, in 2012, the incidence of obesity and diabetes was dramatically increasing in rapidly developing countries such as India, Brazil, China and Middle East countries [7]. In such year, the WHO gave a warning in this aspect, after diabetes caused 1.5 million of deaths; more than 80% of them occurred in low- and middle-income countries (http://www.who.int/mediacentre/fact-sheets/fs312/en/).

The hypothesis explaining such effect, arising from the concept of the Developmental Origin of Health and Disease (DOHaD; [8]), would be related to interactions among genetic background, prenatal environment and postnatal lifestyle of the individuals [9,10]. Humans living in developing countries are characterized by intrinsic ethnic features, with ancestors adapted to food scarcity and, therefore, having developed thrifty genotype for surviving in such conditions. However, they are currently exposed to abundant caloric food and low physical activity [11,12,13,14,15]. The interactions between genetic traits and lifestyle have been classically studied during periods of juvenile development and adulthood but the paramount importance of prenatal environment is nowadays fully acknowledged. Nevertheless, the research on the relationship between genetics and prenatal environment makes necessary to undertake interventional and invasive studies that cannot be carried out in humans. Hence, research in translational animal models is mandatory; preferable in models more similar to humans than rodents, so their predictive value can be improved [16,17,18,19,20]. 

The pig is currently considered a unique model for nutrition-related pregnancy pathologies [21] with the relative role of genetic and environmental factor on postnatal traits having been extensively studied in pigs [22,23,24,25]. The swine model has the advantage of sharing numerous and essential similarities with human beings: omnivorous habits, propensity to sedentary behavior and obesity, as well as similar metabolic, gastrointestinal and cardiovascular features [26,27,28,29,30]. Serum lipid patterns of obese pigs and humans are similar and earliest studies on obesity and pregnancy have shown that triglycerides concentration and distribution of cholesterol between the lipoprotein fractions was similarly modified in mothers and fetuses [31].

Moreover, there is a specific breed, the Iberian pig, which is an amenable model for studies in obesity and associated diseases [32,33]. Most of the studies have been performed in mice in which the most abundant forms of monogenic obesity in humans have been replicated [34]. Main mouse models for obesity studies are based in mutations in genes encoding leptin (Lep^ob/ob^ mouse) and its receptor (Lepr^db/db^ mouse). However, obesity is commonly polygenic and, then, the Iberian pigs constitute a robust model. The Iberian pig is characterized by an adaptive thrifty genotype, similar to humans, for surviving in harsh environments with food scarcity where the animals have been traditionally reared [35]. These traits include, in comparison to Large White and other commercial cycles, longer production cycles, increased energy accumulation in fat reserves; higher levels of leptin circulating in plasma, structural and functional variations in the leptin receptor (*LEPR*) and gene expression changes in the hypothalamus and muscle [36,37,38,39,40,41]. These “thrifty” metabolic traits mean that any alterations in prenatal environment may affect Iberian pigs more extensively than lean commercial breeds. Hence, the Iberian pig model has been successfully used for studies on DOHaD [42,43,44,45,46]. In brief, the exposure of Iberian sows to overnutrition or undernutrition during pregnancy modifies offspring growth patterns and this effect is clearly modulated by the timing of exposure to malnutrition. Offspring exposed to malnutrition during the entire pregnancy, either by deficiency or excess, are similar in size at birth to offspring developed under adequate nutritional conditions. However, in case of exposure to obesogenic diets during juvenile development, the individuals programmed by prenatal malnutrition markedly increase body corpulence and excess fat accumulation when compared with offspring born from pregnancies with an adequate diet. Moreover, programmed individuals are at risk for metabolic syndrome at very early-life (juvenile) stages and these alterations are worsened, even to reach the prodrome of type-2 diabetes, in the offspring from overfed pregnancies.

However, previous research comparing Iberian and lean pigs to better understand obesity and its effects, the offspring was gestated in their corresponding obese or lean mothers [47,48], so the maternal influence could not be left aside. The aim of the present study was to elucidate the importance of offspring genotype on postnatal development, independently of confounding factors related to prenatal (maternal) environment and postnatal lifestyle. The Iberian model allows us to determine such relative roles of genetics and environment by comparing two genotypes (purebred Iberian and crossbreds Iberian × Large White), produced in one single maternal environment (pure Iberian mothers) through artificial insemination of Iberian sows with Iberian and Large White heterospermic semen [49,50] and maintained in the same conditions during postnatal development. 

## 2. Materials and Methods

### 2.1. Ethic Statement

The experiment was assessed and approved by the Instituto Nacional de Investigación y Tecnología Agraria y Alimentaria (INIA) Committee of Ethics in Animal Research (report CEEA 2013/036) on 30th March 2016 and subsequently by the regional competent authority (report PROEX114/16) on 4th May 2016, according to the Spanish Policy for Animal Protection (RD 53/2013) which meets the European Union Directive 2010/63/UE on the protection of research animals. 

### 2.2. Animals and Experimental Design

The study involved 143 piglets born from 16 purebred Iberian sows (mean body-weight of 124.9 ± 3.7 kg) at the farm Ibericos de Arauzo 2004 S.L. (Zorita de la Frontera, Salamanca, Spain). Pregnancies were obtained after cycle synchronization with altrenogest (Regumate, MSD, Boxmeer, The Netherlands) and insemination with heterospermic seminal doses achieved by mixing semen from two purebred Iberian (183 and 197 kg of body-weight) and two purebred Large White boars (324 and 352 kg of body-weight). In brief, immediately after collection, the ejaculates obtained from these males were evaluated for semen quality (sperm concentration, morphology and motility), mixed at equal viable spermatozoa concentrations for Iberian and Large White fractions and aliquoted into 80 mL doses containing 6 × 10^9^ viable spermatozoa. All sows and boars were previously genotyped by pyrosequencing to confirm homozygosity for *LEPRc.1987T* (for the Iberian genotype) and *LEPRc.1987C* (for the Large White genotype), as previously described [28]. The sows were fed a standard grain-based diet (89.9% of dry matter, 13% of crude protein, 2.6% of fat and 2.2 Mcal/kg of metabolizable energy) adjusted to fulfill individual pregnancy and lactation requirements based on data from the National Research Council [51]. 

At birth, the total number of piglets (both alive and stillborn) was recorded for each sow (mean litter size 9.3 ± 2.0 piglets). Sex, weight and head and body measurements (biparietal diameter, crown-rump length, trunk length and abdominal and thoracic circumferences) were recorded for each piglet at birth. All living piglets were sampled for ascertaining homo- or heterozygosity for the *LEPR* gene and tagged with earrings for their identification. After that, piglets underwent fostering to equalize the number of them among sows. A total of 104 piglets were purebred Iberian (IB × IB; 51 females and 53 males) whilst 39 were crossbred Iberian × Large White (IB × LW; 20 females and 19 males). All these piglets remained with sows in individual pens until weaning at the age of 21 days-old, when they were moved to collective pens and fed with a standard diet (89.5% of dry matter, 15% of crude protein, 4% of fat and 2.4 Mcal/kg of metabolizable energy; Table S1) adjusted to fulfill growing requirements. At 60 days-old, a representative group of 67 piglets were selected and sampled (*n* = 36) or maintained at INIA facilities (*n* = 31) for the assessment of early (60–150 days-old) and late juvenile development (210 days-old). During this second growing phase, the animals were fed a diet a standard diet (89.7% of dry matter, 12% of crude protein, 3.5% of fat and 2.4 Mcal/kg of metabolizable energy; Table S1).

### 2.3. Assessment of Morphological and Homeostatic Features of Piglets during Early Postnatal Development

All the piglets were weighted again at the age of 21 (weaning) and 60 days-old (completion of early growing phase). Weight values were used to determine the evolution of the Average Daily Weight Gain (ADWG; weight gained per day) and the Fractional Growth Rate (FGR; weight gained per day per starting weight) for the time-intervals. At 60 days-old, 22 IB × IB (9 females and 13 males) and 14 IB × LW (7 females and 7 males) piglets were sampled to ascertain the effects of the genotype and sex on early-postnatal body-weight and -size, adiposity, body composition, plasma indexes of oxidative stress and antioxidant capacity, plasma parameters of different metabolic pathways and fatty acids composition of subcutaneous fat, muscle and liver.

### 2.4. Assessment of Morphological and Homeostatic Features of Piglets during Juvenile Development

From 60 to 150 days-old, assessment of growth patterns, adiposity and metabolism during early juvenile development was performed monthly in the remaining 31 piglets (18 IB × IB, 9 females and 9 males and 13 IB × LW piglets, 6 females and 7 males). Once a month, all the pigs were weighted. These values were used to determine the evolution of ADWG and FGR monthly and during lifetime. Concomitantly, loin diameter and subcutaneous back-fat depth (total back-fat and both inner and outer layers separately)were measured at the P2 point (located at 4 cm from the midline and transversal to the head of the last rib) with a multifrequency linear-array ultrasonographic probe (SV1 Wireless scanner, SonopTek, Beijing, China). Outer and inner layers of backfat were assessed separately, having in mind that the outer layer is more related to thermoregulation, whereas the inner layer is more metabolically active [52]. At 120 and 150 days-old, blood samples were drawn from the orbital sinus using sterile 10-mL EDTA vacuum tubes (Vacutainer^®^ Systems Europe, Becton Dickinson, Meylan Cedex, France) after fasting for approximately 16 h. The samples were centrifuged at 1500 *g* for 15 min and the plasma was stored in polypropylene vials at −80 °C until assayed for determination of different plasma and metabolic parameters. These 31 piglets were measured and sampled again at 210 days-old to determine body weight, size and composition, plasma indexes of oxidant/antioxidant and metabolic status and fatty acid composition of subcutaneous and visceral fat, muscle and liver at late juvenile development.

### 2.5. Evaluation of Body Composition and Organs Weight of Piglets at 60 and 210 Days-Old

At both 60 and 210 days-old samplings, pigs were euthanized by stunning and exsanguination in compliance with standard procedures (RD 53/2013), with a blood sample being previously drawn and processed as formerly described, to determine plasma indexes of oxidant/antioxidant and metabolic status. Immediately, body measures (biparietal diameter, occipito-nasal length, trunk length and thoracic and abdominal circumferences) and back-fat depth and loin diameter were recorded. Afterwards, the head was separated from the trunk at the atlanto-occipital joint and, after recording the ratio of head to body weight, the brain was extracted from skull and weighed. Then, all thoracic and abdominal viscera were removed and weighted together. Finally, the major organs (heart, lungs, liver, intestine, kidney, spleen, pancreas and adrenal glands) were weighed individually. The following weight ratios were considered: weights of brain, heart, lungs, liver, kidneys, intestine, pancreas, spleen and adrenal glands relative to total viscera weight.

### 2.6. Evaluation of the Oxidant/Antioxidant Status of the Piglets

Values for total antioxidant capacity were assayed in the plasma samples obtained at 60 and 210 days-old, by using the ferric reducing antioxidant power assay (FRAP; [53]) as previously described. Assessment of lipid peroxidation was performed in the same samples by measuring malondialdehyde (MDA) using the thiobarbituric acid reaction [54].

### 2.7. Evaluation of the Metabolic Status of Piglets

Plasma indexes for metabolism of glucose, lipids and proteins were determined in the plasma samples obtained at 60, 120, 150 and 210 days-old. The glycemic profile was assessed by determining plasma glucose and fructosamine concentrations. Lipids metabolism was evaluated by determining plasma concentrations of total cholesterol, high- and low-density lipoproteins cholesterol (HDL-c and LDL-c, respectively) and triglycerides. Protein metabolism was assessed by determining urea and, finally, metabolic state was also assessed through measuring plasma lactate. All metabolites were determined using a clinical chemistry analyzer (Konelab 20, Thermo Scientific, Vantaa, Finland), according to the manufacturer’s instructions.

### 2.8. Evaluation of the Fat Content and Fatty Acid Composition of Tissue Samples

The fat content and fatty acids composition in the piglets were determined in samples, obtained immediately after euthanasia, of subcutaneous fat (plus visceral fat at 210 days-old), *longissimus dorsi* (LD), *biceps femoris* (BF) and liver. Intramuscular and liver fat were extracted as described by Segura et al. [55] after lyophilization and homogenization; fat content in each tissue was calculated and expressed as a percentage. The neutral lipid fraction (triglycerides) and the polar lipid fraction (phospholipids) were separated using aminopropyl minicolumns previously activated with 7.5 mL of hexane [56]. Subcutaneous and visceral fat were extracted directly. In the case of back-fat, outer and inner layers were analyzed separately to ascertain possible differences [52]. The fatty acids composition of all tissues was analyzed using gas chromatography [57]. The quantities of individual fatty acids expressed as g/100 g of total fatty acid content were used to calculate the proportions of saturated fatty acids (SFA), monounsaturated fatty acids (MUFA), polyunsaturated fatty acids (PUFA) and total n3 and n6 FA (N3 and N6, respectively), as well as the ratios N6/N3 and MUFA/SFA and the unsaturation index (UI; calculated as follows: 1 (% monoenoics) + 2 (% dienoics) + 3 (% trienoics) + 4 (% tetraenoics) + 5 (% pentaenoics) + 6 (% hexaenoics) [58,59]. Furthermore, the desaturation index (DI) was used to determine the activity of the stearoyl-CoA desaturase enzyme 1 (SCD1; ratio of the enzyme product, MUFA mainly oleic acid [C18:1n-9], to the enzyme substrate, SFA mainly stearic acid [C18:0]; [60]). Finally, the activities of the desaturase enzymes for N6 (DN6) and N3 (DN3) were estimated from the ratios C20:4n6/C18:2n6 and C20:5n3/C18:3n3, respectively. ∆9 desaturase activity (D9) was estimated from the ratio (C16:1n7 + C18:1n9)/(C16:1n7 + C18:1n9 + C18:0 + C16:0).

### 2.9. Statistical Analysis

Data were analyzed using SPSS^®^ 25.0 (IBM, Armonk, New York, NY, USA). Dependent variables related to offspring phenotype (weight, ADWG, FGR, back-fat depth, loin diameter, organ weights, indexes of metabolic state and oxidative stress and fatty acids composition) were assessed using two-way ANOVA in a General Linear Model; interactions among independent variables (offspring genotype, offspring sex and their interaction) were observed and fixed when statistically significant. Changes over time in weight and measures were assessed by ANOVA for repeated measures with the Green-Houser-Geisser correction when statistically significant. The piglet was the experimental unit. All the results were expressed as mean ± S.E.M. Statistical significance was accepted from *p* < 0.05 and a statistical trend was considered when 0.05 < *p* < 0.1.

## 3. Results

### 3.1. Effects of Genotype and Sexon Litter Characteristics and Prenatal Development of the Piglets

**Figure 1:**
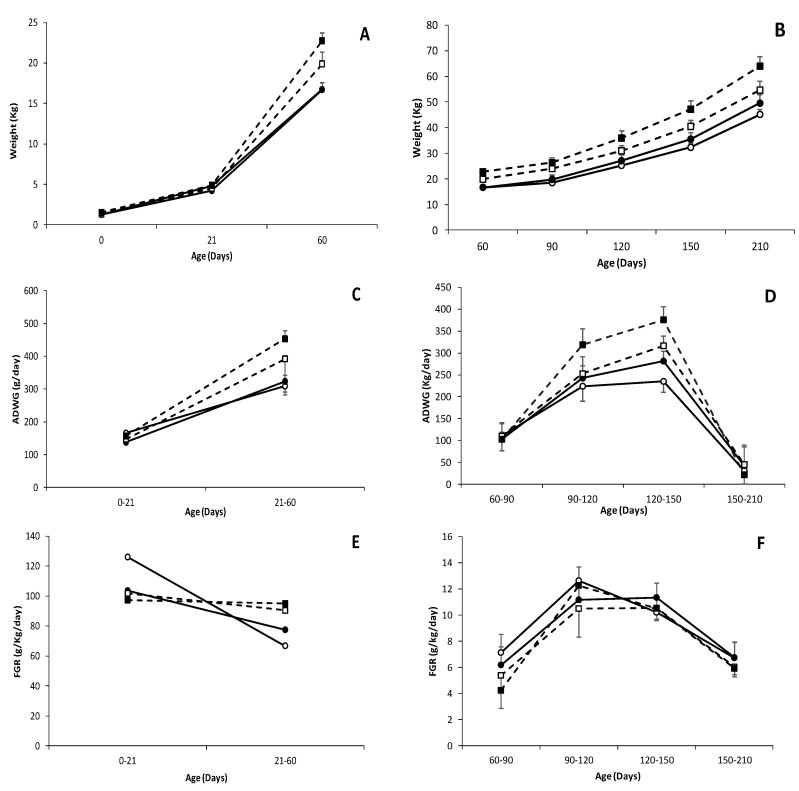
Changes over time in mean values (±S.E.M.) of body weight (**A**,**B**), Average Daily Weight Gain (ADWG); (**C**,**D**) and Fractional Growth Rate (FGR); (**E**,**F**) during early postnatal (0 to 60 days-old; left panels) and juvenile periods (60 to 210 days-old; right panels), in female and male (white and black dots, respectively) pure Iberian (IB × IB; continuous line with circle dots) and Iberian × Large White crossbreds (IB × LW; discontinuous line with square dots).

The birth weight and size of the piglets were significantly different between genotypes. Values are detailed in Figure 1 and Tables S2 and S3. The IB × LW piglets were heavier at birth than their IB × IB counterparts (1.4 ± 0.4 vs. 1.3 ± 0.3 kg, respectively; *p* < 0.05). The values for trunk length were also higher in IB × LW newborns (24.0 ± 2.7 vs. 22.6 ± 2.2 cm in IB × IB; *p* < 0.01), while abdominal and thoracic circumferences were similar between genotypes and occipito-nasal length was greater in IB × IB (11.2 ± 0.5 vs. 10.9 ± 0.8 cm in IB × LW; *p* < 0.05).

Effects from sex are detailed at Tables S2 and S3. Differences in body weight at birth were especially relevant between males (1.27 ± 0.05 in IB × IB males vs. 1.52 ± 0.10 kg in IB × LW males; *p* < 0.05) whereas no significant differences were found in females of different genotype. Sex also affected the occipito-nasal length, showing a trend to be larger in IB × IB males than in IB × LW males (11.3 ± 0.09 vs. 11.0 ± 0.17 cm, respectively; *p* = 0.091) and the trunk length, which was significantly shorter in IB × IB males than in IB × LW males (22.6 ± 0.35 vs. 24.5 ± 0.59 cm, respectively; *p* < 0.05).

### 3.2. Effects of Genotype and Sex on Postnatal Patterns of Growth and Development of Piglets

There were no significant differences between genotypes in the Average Daily Weight Gain (ADWG; 151.0 ± 4.8 for IB × IB and 152.0 ± 9.5 g/day for IB × LW) during the suckling period (0–21 days-old; Figure 1) but IB × IB piglets showed a significantly higher Fractional Growth Rate during this period (FGR; 115.0 ± 3.5 vs. 99.4 ± 4.3 g/kg/day in IB × LW; *p* < 0.05). There were no significant differences between genotypes in body weight at weaning but a genotype-by-sex interaction was found (*p* < 0.05), with IB × IB females and IB × LW males being heavier than their same genotype counterparts. The same effects were found when assessing body size (biparietal diameter and thoracic and abdominal circumferences; *p* < 0.05 for all). 

After weaning, with intake of solid grain-based diets from 21 to 60 days, both ADWG and FGR were significantly higher in the IB × LW group with these piglets being therefore heavier (*p* < 0.001) and larger (*p* < 0.05 for all body measures) at 60 days-old. These differences, excepting a similar FGR, were maintained during all the juvenile development and IB × LW pigs continued being heavier (*p* < 0.001 at all time-points) and larger (*p* < 0.05 for all body measures, excepting for their occipito-nasal length at 210 days old) than the IB × IB offspring.

There were significant sex-related effects on the body size of the pigs, especially from 60 days-old onwards. During this period, the values of all measures were higher in IB × LW than in IB × IB males, excepting occipito-nasal length (*p* < 0.05 for all of them). In the females, only the trunk length was significantly longer in the IB × LW group (88.8 ± 0.98 vs. 96.5 ± 1.73 cm; *p* < 0.01). Regarding body weight, differences between genotypes were more prominent between males at 60 days-old (*p* < 0.001). From 90 days-old onwards, body weights were similar between females and males of both genotypes (*p* < 0.05 for both sexes at all ages). Assessment of ADWG and FGR showed significant differences in ADWG between 21 and 60 days-old in males (*p* < 0.0005; higher values in IB × LW) but the differences were non-significant between females. Finally, both ADWG and the FGR were lower in IB × IB males when compared with IB × IB females (*p* < 0.01) between 21 and 60 days-old. 

### 3.3. Effects of Genotype and Sex on Body Composition, Muscle Accretion and Adiposity

**Figure 2:**
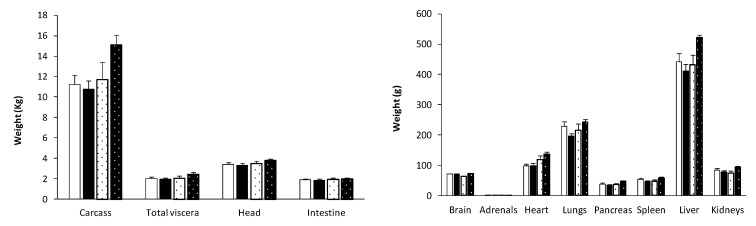
Mean weight ± S.E.M. of the major organs in pure Iberian (IB × IB; solid bars) and Iberian × Large White crossbred piglets (IB × LW; dotted bars), either females or males (white or black background, respectively), at the age of 60 days-old.

**Figure 3:**
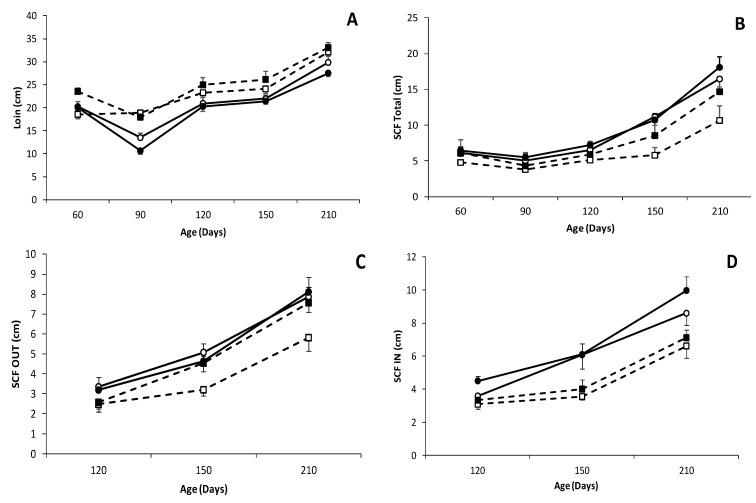
Changes over time in mean values (± S.E.M.) of loin diameter (**A**) and depth of total subcutaneous back-fat (SCF); (**B**) and of its inner and outer layers (SCF IN and OUT); (**C**,**D**) respectively during juvenile development (60 to 180 days-old; lower panels) in female and male (white and black dots, respectively) pure Iberian (IB × IB; continuous line with circle dots) and Iberian × Large White crossbreds (IB×LW; discontinuous line with square dots).

**Figure 4:**
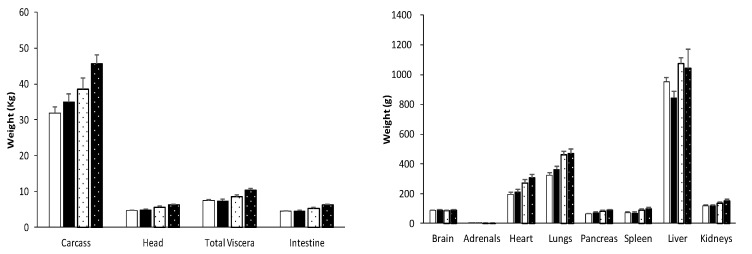
Mean weight ± S.E.M. of the major organs in pure Iberian (IB × IB; solid bars) and Iberian × Large White crossbred piglets (IBLW; dotted bars), either females or males (white or black background, respectively), at 210 days-old.

There was a trend for a higher carcass weight in IB × LW piglets at 60 days old (13.4 ± 1.0 vs. 11.2 ± 0.6 kg in IB × IB, *p* = 0.066; Figure 2 and Table S4); such difference being significant when comparing males (15.1 ± 1.0 kg in IB × LW vs. 10.8 ± 0.8 kg in IB × IB; *p* < 0.005). The study of muscle development (assessed as loin diameter) showed higher values in IB × LW animals during all the period of study, with significant differences from 90 days-old onwards (Figure 3) and therefore with a higher carcass weight at 210 days-old (42.4 ± 8.9 vs. 33.4 ± 1.4 kg in IB × IB, *p* < 0.05; Figure 4 and Table S5).

Conversely, the estimation of adiposity (assessed as subcutaneous fat depth, intramuscular at the *longissimus dorsi* and *biceps femoris* and intrahepatic fat) showed a larger total backfat depth in the IB × IB group at all time-points (*p* < 0.05; Figure 3) and in both the outer and inner layers from 120 days-old onwards; (*p* < 0.05). Adiposity differences between groups were more pronounced when comparing females from 150 days onward (*p* < 0.01 at 150 days-old and *p* < 0.05 at 210 days-old, higher in IB × IB females than IB × LW females), whereas no significant differences were found between males. Significant differences at the *biceps femoris* intramuscular fat were found at 60 days-old and 210 days-old (8.2 ± 1.3% in IB × IB vs. 7.1 ± 1.6% in IB × LW and 16.1 ± 4.4% in IB × IB vs. 12.2 ± 2.9% in IB × LW, respectively; *p* < 0.05 for both). Intramuscular fat at *longissimus dorsi* showed a trend to be higher in IB × IB pigs at 210 days-old (15.3 ± 7.1 vs. 11.9 ± 3.2% in IB × LW; *p* = 0.09).

The assessment of body composition at 60 days-old (Figure 2 and Table S4) showed a trend for higher values of head weight in IB × LW males than in IB × IB males (2.4 ± 0.2 kg vs. 2.0 ± 0.1 kg, respectively; *p* = 0.056), with significant differences when comparing brain/head weight-ratio (0.04 ± 0.0 for IB × IB vs. 0.03 ± 0.0 for IB × LW; *p* < 0.05). Similarly, values for brain weight also showed a trend for interaction between genotype and sex (higher values in IB × IB females than in IB × IB males and in IB × LW males than in IB × LW females; *p* = 0.055). Although overall weight of both thoracic and abdominal organs did not differ between genotypes, significant differences in some individual organs were found when only comparing males for some individual organs. Thus, IB × LW males showed higher weights of heart (136 ± 7.5 vs. 98.9 ± 7.1 g, *p* < 0.01), liver (520.5 ± 9.8 vs. 412.3 ± 21.1 g, *p* < 0.01), kidneys (93.3 ± 3.3 vs. 78.46 ± 4.4 g, *p* < 0.05), pancreas (46.9 ± 3.0 vs. 34.3 ± 2.6 g, *p* < 0.01) and spleen (58.29 ± 3.4 vs. 46.15 ± 3.3 g, *p* < 0.05). 

At 210 days old (Figure 4 and Table S5), the head was significantly heavier in the IB × LW pigs (5.9 ± 0.7 vs. 4.7 ± 0.7 kg for IB × IB; *p* < 0.001) but, conversely, brain-to-head weight ratio was significantly higher in IB × IB (0.019 ± 0.003 vs. 0.015 ± 0.003 for IB × LW; *p* < 0.001). Total and individual weights of thoracic and abdominal viscera, excepting that of the adrenal glands, were higher in IB × LW pigs (*p* < 0.001 for total and *p* < 0.01 for individual weights). However, differences in weight-ratios were only significant for the relative weight of heart (0.030 ± 0.004 in IB × LW vs. 0.027 ± 0.005 in IB × IB; *p* < 0.05). There were sex-related differences in the relative weights of lungs (higher in IB × IB males and IB × LW females than in their counterparts; *p* < 0.05) and liver (higher in females in both genotypes; 0.13 ± 0.01 vs. 0.12 ± 0.01; *p* = 0.051 for IB × IB and 0.13 ± 0.01 vs. 0.10 ± 0.03 *p* = 0.57 for IB × LW).

### 3.4. Effects of Genotype and Sex on the Antioxidant Capacity and Oxidative Stress of Piglets

The total antioxidant capacity at 60 days old was affected by a genotype-by-sex interaction (Table 1), with IB × LW males showing significantly higher values than the IB × LW females (*p* < 0.005) whereas IB × IB females showed the higher values in their genotype (although the difference was not significant). At this age, lipid peroxidation was significantly higher in the IB × LW group (*p* < 0.05) and higher when comparing IB × LW from IB × IB females (*p* < 0.05). However, there were no differences when comparing genotypes or sexes at 210 days old.

### 3.5. Effects of Genotype and Sex on Metabolic Status of Piglets

The metabolic status of the pigs was affected by genotype and sex (Table 2 and Table S6). The lipid profile was mainly affected by genotype, with IB × IB offspring showing higher plasma concentrations of total cholesterol than IB × LW counterparts from 120 days-old onwards (*p* < 0.01 at 120 and 150 days-old and *p* < 0.05 at 210 days-old); plasma concentrations of HDL-c and LDL-c were also significantly higher in IB × IB at 120 and 150 days old (*p* < 0.01 for HDL-c and *p* < 0.05 for LDL-c). In the same way, triglycerides were higher in IB × IB animals with significant differences at 150 and 210 days old (*p* < 0.01). There were no significant effects from sex and a trend for higher plasma total cholesterol in IB × LW males than in their female counterparts at 60 days-old (*p* = 0.069). 

The glycemic profile showed an interaction between genotype and sex in plasma fructosamine concentrations at 60 days-old, with higher values in IB × IB males and IB × LW females than in their counterparts (*p* < 0.05 for both). Values for plasma glucose and fructosamine concentrations did not differ among genotypes and sexes during the juvenile development, excepting a higher glucose concentration in IB × IB than in IB × LW animals at 150 days-old.

The urea concentrations fluctuated over time and were affected by an interaction between genotype and sex. There were higher values in IB × IB than IB × LW males at 60 days old (*p* < 0.05), in IB × IB than IB × LW females at 120 days old (*p* < 0.05) and in IB × IB females and IB × LW males than in their counterparts at 210 days-old (*p* < 0.05 for both). Finally, plasma lactate concentrations were only influenced by genotype at 60 days-old, with higher values in IB × LW pigs (*p* < 0.05).

### 3.6. Effects of Genotype and Sex on Fatty Acid Composition of the Piglets

The fatty acid composition of fat depots and organs was significantly affected by the genotype and sex, with most of the differences being similar between 60 and 210 days-old (Table 3 and Table 4 and Tables S7–S25). 

The assessment of the subcutaneous fat showed that, at both the outer and the inner layers and at both 60 and 210 days-old, purebred IB × IB offspring showed lower amounts of PUFA and N6 (*p* < 0.0005 for both layers and ages in both parameters) and N3 fatty acids (*p* < 0.01 for both layers at 60 days-old and the outer layer at 210 days-old; *p* < 0.05 for the inner layer at 210 days-old). The IB × IB pigs also showed a lower unsaturation index at both the outer and the inner layers and at both 60 and 210 days-old (*p* < 0.01 for both layers and ages). At 60 days-old, purebred IB × IB offspring showed higher amounts of MUFA at both the outer and the inner layers (*p* < 0.01), while such difference was only significant at the outer layer at 210 days-old (*p* < 0.01). At this age, the inner layer of IB × IB animals showed a higher amount of SFA (*p* < 0.05). Finally, IB × IB showed a higher DI and D9 activity at the inner layer at 60 days-old (*p* < 0.05 for both). Their DN6 activity was higher at the inner layer (*p* < 0.01) and lower at the outer layer than in IB × LW pigs (*p* < 0.05). Sex had an important effect on the fatty acid composition of the subcutaneous backfat at both ages. Thus, the outer and inner layers of the subcutaneous fat showed more differences between females than between males. In the inner and outer layer, MUFA (*p* < 0.01 for the outer layer and *p* < 0.0005 for the inner layer at 60 days-old; *p* < 0.0005 for the outer layer and *p* = 0.065 for the inner layer at 210 days-old), MUFA/SFA (*p* < 0.05 for the outer layer at 60 days-old and *p* < 0.01 for the inner layer at both ages), D9 and DI (*p* < 0.05 for the outer layer and *p* < 0.01 for the inner layer at both ages) were higher in IB × IB females, whereas PUFA and UI were augmented in IB × LW females (*p* < 0.05 for UI and *p* < 0.0005 for PUFA at both layers and ages). Only PUFA were different between males in both layers (*p* < 0.01 for the outer layer and *p* < 0.05 for the inner for all parameters at both ages).

The data obtained after the analysis of the visceral fat at 210 days-old resembled the results from subcutaneous fat, especially at the inner layer. IB × IB pigs showed lower amounts of PUFA, N6 and N3 (*p* < 0.0005 for all), a lower unsaturation index (*p* < 0.01) and a higher content of SFA and DN6 activity (*p* < 0.05 and *p* < 0.01, respectively). In IB × IB females, higher levels of MUFA and in IB × LW females higher levels of PUFA were observed (*p* < 0.01 for both). However, between males only the PUFA content was different (*p* < 0.05). Between sexes within each genotype, some differences were found in the outer and inner layer of the subcutaneous fat at 210 days-old. IB × IB males had lower UI, MUFA/SFA (*p* < 0.05), D9 and DI (*p* < 0.01) when compared with their female counterparts in both layers. However, in the inner layer, IB × IB males had higher levels of SFA. In the IB × LW group, females had higher levels of MUFA, PUFA and UI (*p* < 0.05 except for PUFA; *p* < 0.01).

There were few changes, at both 60 and 210 days-old, in the fatty acid composition of the *longissimus dorsi* muscle and only at the polar fraction. At 60 days-old, IB × IB piglets showed higher content of PUFA and N6 and thus a higher N6/N3 ratio (*p* < 0.05 for all). These differences were lost at 210 days-old, when only a higher DN3 activity in the IB × IB pigs was found (*p* < 0.05). Sex affected the polar fraction at 60 days-old, specifically the N3/N6 ratio, which was significantly higher in IB × IB males when compared with the IB × LW males (*p* < 0.05) and the neutral fraction at 210 days-old, being N6 content significantly higher in IB × LW males when compared with IB × IB males. 

Differences were more evident at the *biceps femoris* and mainly at the polar fraction, where the N6/N3 ratio was higher in IB × IB pigs (*p* < 0.05 at both 60 and 210 days-old). At 210 days-old, the polar fraction of IB × IB pigs showed higher MUFA content and MUFA/SFA ratio and lower unsaturation index (*p* < 0.01 for all) and lower N3 amount and DN3 activity and higher D9 activity (*p* < 0.05 for all). On the other hand, the neutral fraction of the *biceps femoris* of IB × IB piglets showed a higher amount of N3 and a lower DN3 activity at 60 days-old (*p* < 0.05 for both) and a higher DN6 activity at 210 days-old (*p* < 0.0005). However, when the sex effect was studied, it was more obvious at the neutral fraction at 60 days-old, with a more intense effect being observed in females than in males. Thus, IB × IB females showed higher MUFA, MUFA/SFA, DI and D9 than the IB × LW females (*p* < 0.05 for all), whereas at this age the polar fraction showed differences only between males in the N6/N3 ratio (*p* < 0.05; higher in IB × IB) and in the SFA content only between females (*p* < 0.01; higher in IB × IB). When sexes within each genotype were studied, differences between males and females in the IB × LW group were found. The values of MUFA and MUFA/SFA were higher in males, whereas PUFA and UI were higher in females (*p* < 0.05 except for PUFA; *p* < 0.01). There were also differences in D9 and DI activity, being higher in males (*p* < 0.05 for both). 

There were, conversely, very few differences between genotypes in the fatty acid composition of the liver at both 60 and 210 days-old. The desaturation index (DI) at the neutral fraction was significantly higher in IB × LW animals at 60 days-old (*p* < 0.05), whereas only a trend for a higher amount of N3 in the neutral fraction of IB × IB pigs (*p* = 0.085) was found at 210 days-old. The same accounted for sex-related effect, with only higher values of DI and DN3 at the neutral fraction in IB × LW males than in IB × IB males at 60 days-old (*p* < 0.05 for both).

### 3.7. Overview of Interactions between Genotype and Sex on Fatty Acid Composition of the Piglets

The genotype × sex interactions found in the different parameters previously defined and detailed in Supplementary tables, as a synopsis of the main findings in the study, are summarized in the Table 5.

## 4. Discussion

The model used in the present study, comparing the postnatal development of purebred (IB × IB) and crossbred (IB × LW) littermates born from purebred IB × IB sows, allows us to clearly discern phenotype traits driven by the genotype from features imposed by the pre- and postnatal environment. The results obtained in this study support the well-known relevance of genotype but also evidence a paramount role of the interaction sex-by-genotype, with differential effects depending on the offspring genotype and sex.

In brief, birth-weight and -size of the piglets were significantly different between genotypes, supporting previous data obtained at prenatal stages in the same model [49]. The IB × LW piglets were heavier and longer, whilst abdominal and thoracic circumferences were similar and head was longer in IB × IB offspring. Afterwards, IB × LW piglets were always heavier and larger than IB × IB piglets, with better carcass and muscle development; conversely, IB × IB piglets showed a higher adiposity. These findings are similar to those obtain in previous studies comparing purebred Large White and Iberian genotypes [61,62,63,64] and, taking into account the proper characteristics of the breeds, are logical: Large White pigs have been selected for higher body size and meat content during decades whilst the Iberian pig is a rustic breed with scarce selection. In fact, these were the reasons for choosing both genotypes in the model and the present study. 

The higher adiposity and the leptin-resistant genotype of the Iberian breed, with predisposition to insulin resistance and dyslipidemia [32,33], were also evident in the IB × IB offspring, which showed high plasma levels of fructosamine, cholesterol and triglycerides. Differences in fat deposition were accompanied by differences in fatty acid composition since, in mature pigs, adipose tissue is the major site of fatty acid synthesis [65] and its composition is influenced by genotype, sex, nutrition and other environmental factors [66]. In the present study, differences were mainly found at subcutaneous and visceral fat, the adipose tissues which are metabolically more active [67,68].

The IB × LW piglets showed a higher PUFA content (both N3 and N6 PUFA) and a lower MUFA content, which is a result of the selection of commercial breeds (it is the case of the Large White breed) for reduced fat accumulation and lipogenesis, explaining the lower concentration of MUFA and SFA observed and, therefore, a higher PUFA content [66]. Furthermore, IB × IB pigs had a higher desaturation index (DI), which represents the activity of the stearoyl-CoA desaturase enzyme 1 (SCD1). In both human and pigs, increased desaturation index and SCD1 activity have been related to metabolic disorders, like alterations in lipogenesis and insulin regulation [69,70,71,72].

On the other hand, the fatty acid composition of the non-adipose tissue (i.e., muscle) showed a lower content of N3 PUFA and a higher content of N6 PUFA and, thus, a higher N6/N3 ratio in IB × IB piglets, in agreement with previous studies at fetal stages [49]. The values of N3 PUFA have been related with a healthier status, due to its role in anti-inflammatory function, in both humans [73] and pigs [74], whilst N6 PUFA have been related to a pro-inflammatory state [75]. Hence, our results in the IB × IB individuals may indicate evidences of a chronic low-grade inflammation, as described in human obesity [76,77].

These differences in body weight and size, metabolic traits and fatty acids composition between IB × IB and IB × LW piglets were more evident when comparing sexes within genotypes. First evidences were found when assessing the early postnatal development, when IB × IB piglets had a significantly higher Fractional Growth Rate (FGR) during the suckling period, which indicates a catch-up growth in this breed. At weaning, as a result of a genotype-by-sex interaction, IB × IB females and IB × LW males were heavier than their within-genotype counterparts, as previously described [78,79,80]. These sex-related effects were maintained during all the period of study and IB × IB males remained smaller and lighter than IB × LW males whilst IB × IB females showed similar size and weight to IB × LW females. Such increased growth in female than in male IB × IB fetuses supports earlier data obtained in this swine breed [39,79].

The importance of the offspring sex in the developmental programming is widely known and differences between sexes have been early related to the different ability of male and female fetuses to respond to an environmental challenge [80]. However, there is also an alternative hypothesis which proposes that, given that programming interventions are mediated by maternal physiology, all fetuses would respond identically to the same insult but their “opportunity to respond” would be buffered by the mother depending on the offspring sex [80]. In fact, pregnancy is a state of equilibrium between the necessities of mother and progeny. As a result, mothers do not invest more than indispensable in their offspring growth, as a preventive strategy for safeguarding their own energetic balance during and after pregnancy and even for future gestations [81,82]. Evolutionary biology traditionally views males as the “most expensive and less useful” sex, so may therefore not be energy efficient for a mother to heavily invest in male offspring when the investment in adequate development and homeostasis of female progeny would guarantee the chances to reproduce and maintain the species through gene transmission to future generations [83,84].

The model used in this study and the different results obtained in IB × IB and IB × LW males and females suggest that sex-related differences in developmental patterns are not common to all offspring independently of breed and species but strongly modulated by the offspring genotype. In this regard, our results indicate that the protective effects described for female fetuses are exacerbated in the thrifty genotypes or, may be, alleviated in lean genotypes not exposed to environmental challenges. Analogous results were previously found when comparing purebred IB × IB and LW × LW offspring gestated in purebred IB × IB and LW × LW, respectively [48] but the current study undoubtedly evidences that these effects are driven by the offspring genotype independently of maternal influences.

Hence, the translational result to human medicine of the present study is that both preclinical and clinical studies on pregnancy and offspring development need to consider the genotype, (ethnicity), of the individual.

## 5. Conclusions

The present study supports the differential roles of genotype and sex on offspring phenotype (i.e., growth and development, metabolic and antioxidant status and fatty acid composition of different tissues) independently of the pre- and postnatal environment. These results are highly interesting to understand underlaying differences between ethnicities, which may set the basis for future research.

## Figures and Tables

**Table 1:** Mean plasma concentrations (± S.E.M.) for parameters related to total ferric antioxidant capacity (FRAP) and lipid peroxidation (malondialdehyde; MDA) in female and male pure Iberian (IB × IB) and Iberian × Large White crossbred pigs (IB × LW) at 60 and 210 days-old.

Parameter	IB × IB				IB × LW			Total	F vs M		IB × IB vs IB × LW		Gen × Sex
	Age (Days)	Total	Females	Males	Total	Females	Males		IB × IB	IB × LW	F	M	
FRAP (µmol/mL)	60	17.4 ± 1.76	18.0 ± 3.35	16.9 ± 1.97	26.9 ± 6.10	10.3 ± 1.88	31.7 ± 5.01	NS	NS	***	+	+	***
	120	24.8 ± 1.88	25.0 ± 2.38	24.6 ± 3.05	22.9 ± 1.64	18.9 ± 1.85	26.8 ± 0.98	NS	NS	*	NS	NS	NS
MDA (µmol/L)	60	64.1 ± 12.2	62.6 ± 10.2	65.2 ± 13.6	73.1 ± 12.3	77.4 ± 12.3	69.5 ± 12.0	*	NS	NS	*	NS	NS
	120	54.4 ± 6.41	54.3 ± 5.04	54.6 ± 7.87	52.8 ± 4.39	52.8 ± 5.55	52.9 ± 3.58	NS	NS	NS	NS	NS	NS

F = Female; M = Male; Gen = Genotype; + *p* < 0.1; * *p* < 0.05; *** *p* < 0.001; NS = no significant.

**Table 2:** Changes over time in mean plasma concentrations (± S.E.M.) for parameters related to glycemic, lipidemic and protein metabolism in pure Iberian (IB × IB) and Iberian and Large White crossbred pigs (IB × LW) at 60, 120, 150 and 210 days-old. Effects from sex are detailed at Table S6.

mg/dL	60 Days		120 Days		150 Days		210 Days	
	IB × IB	IB × LW	IB × IB	IB × LW	IB × IB	IB × LW	IB × IB	IB × LW
GLU	99.8 ± 4.79	101 ± 4.17	85.1 ± 2.32	89.17 ± 3.80	85.1 ^a^ ± 2.23	95.3 ^b^ ± 5.76	82.6 ± 4.26	84.2 ± 4.02
FRU	222 ± 6.76	226 ± 9.4	222 ± 6.76	227 ± 4.83	227 ± 8.19	241 ± 5.36	252 ± 5.74	254 ± 7.47
CHO	76.6 ± 3.35	82.9 ± 3.29	122 ^c^ ± 3.53	100 ^d^ ± 4.04	124 ^c^ ± 3.75	103 ^d^ ± 3.86	109 ^a^ ± 3.54	95.2 ^b^ ± 5.45
HDL-c	25.6 ± 1.49	29.2 ± 1.59	64.7 ^e^ ± 1.78	53.1 ^f^ ± 2.67	67.0 ^c^ ± 1.62	60.2 ^d^ ± 2.08	55.0 ± 2.46	52.3 ± 6.31
LDL-c	39.5 ± 2.26	45.4 ± 3.27	56.8 ^a^ ± 3.57	41.6 ^b^ ± 1.89	58.2 ^a^ ± 3.70	44.4 ^b^ ± 2.67	48.8 ± 2.67	43.2 ± 3.78
TG	34.6 ± 2.50	34.9 ± 2.18	65.3 ± 3.83	55.0 ± 6.92	64.6 ^c^ ± 4.05	46.8 ^d^ ± 2.50	49.4 ^c^ ± 2.72	34.2 ^d^ ± 2.46
UREA	17.9 ± 1.45	14.7 ± 0.85	27.4 ± 1.16	22.6 ± 2.54	23.8 ± 1.21	22.8 ± 1.52	18.3 ± 1.07	21.6 ± 1.94
LAC	61.1 ^a^ ± 4.41	112 ^b^ ± 5.90	61.1 ± 5.87	54.6 ± 7.83	65.6 ± 5.07	69.6 ± 10.5	64.8 ± 8.44	56.0 ± 7.71

GLU = glucose; FRU = fructosamine; CHO = cholesterol; HDL-c = high density lipoprotein cholesterol; LDL-c = low density lipoprotein cholesterol; TG = triglycerides; URE = urea; LAC = lactate. Different superscripts indicate significant differences a ≠ b *p* < 0.05; c ≠ d *p* < 0.01; e ≠ f *p* < 0.0005.

**Table 3:** Highlight of significant differences (*p* < 0.05) ± S.E.M. in fatty acid composition of the subcutaneous fat, *longissimus dorsi* and *biceps femoris* muscles and liver, at 60 days-old, between pure Iberian (IB × IB) and Iberian × Large White crossbred pigs (IB × LW). Effects from s are detailed at Tables S2–S23.

Tissue	Fraction	Variable	IB × IB	IB × LW
SCF	Out	MUFA (g/100 g)	50.9 ^c^ ± 0.49	48.3 ^d^ ± 0.49
		PUFA (g/100 g)	13.0 ^e^ ± 0.26	15.8 ^f^ ± 0.37
		UI	0.80 ^c^ ± 0.01	0.84 ^d^ ± 0.01
		N3 (g/100 g)	1.13 ^c^ ± 0.03	1.35 ^d^ ± 0.05
		N6 (g/100 g)	11.8 ^e^ ± 0.25	14.5 ^f^ ± 0.34
	In	MUFA (g/100 g)	49.7 ^c^ ± 0.43	47.1 ^d^ ± 0.54
		PUFA (g/100 g)	13.0 ^e^ ± 0.31	15.3 ^f^ ± 0.37
		UI	0.78 ^a^ ± 0.01	0.81 ^b^ ± 0.01
		MUFA/SFA	1.34 ^a^ ± 0.03	1.26 ^b^ ± 0.03
		N3 (g/100 g)	1.06 ^c^ ± 0.03	1.23 ^d^ ± 0.05
		N6 (g/100 g)	11.9 ^e^ ± 0.29	14.1 ^f^ ± 0.34
		D9	0.55 ^a^ ± 0.01	0.53 ^b^ ± 0.01
		DI	3.78 ^a^ ± 0.10	3.38 ^b^ ± 0.12
LD	Polar	PUFA (g/100 g)	43.4 ^a^ ± 0.68	40.2 ^b^ ± 1.60
		N6 (g/100 g)	38.9 ^a^ ± 0.65	36.0 ^b^ ± 1.45
		N6/N3	8.90 ^a^ ± 0.22	8.01 ^b^ ± 0.24
BF	Neutral	N3 (g/100 g)	2.19 ^a^ ± 0.12	3.04 ^b^ ± 0.39
		DN3	0.26 ^a^ ± 0.02	0.35 ^b^ ± 0.05
	Polar	N6/N3	9.99 ^a^ ± 0.21	9.20 ^b^ ± 0.27
Liver	Neutral	DI	0.39 ^a^ ± 0.02	0.46 ^b^ ± 0.03

SCF = subcutaneous fat; LD = *longissimus dorsi*; BF = *biceps femoris*; FA = fatty acids; UI = unsaturation index; MUFA = sum of monounsaturated FA; PUFA = sum of polyunsaturated FA; SFA = sum of saturated fatty acids; DI = desaturation index; D9 = total activity of ∆9 desaturase; DN3 = total activity for the desaturases of n3. Different superscripts indicate significant differences a ≠ b (*p* < 0.05); c ≠ d (*p* < 0.01); e ≠ f (*p* <0.0005).

**Table 4:** Highlight of significant differences (*p* < 0.05) ± S.E.M. in fatty acid composition subcutaneous fat, *longissimus dorsi* and *biceps femoris* muscles and liver, at 210 days-old, between pure Iberian (IB × IB) and Iberian × Large White crossbred pigs (IB × LW). Effects from sex are detailed at Tables S7–S25.

Tissue	Fraction	Variable	IB × IB	IB × LW
SCF	Out	MUFA (g/100 g)	48.4 ^c^ ± 0.30	47.0 ^d^ ± 0.36
		PUFA (g/100 g)	13.6 ^e^ ± 0.30	15.7 ^f^ ± 0.46
		UI	0.78 ^c^ ± 0.01	0.81 ^d^ ± 0.01
		N3 (g/100 g)	1.36 ^c^ ± 0.04	1.55 ^d^ ± 0.05
		N6 (g/100 g)	12.3 ^e^ ± 0.26	14.2 ^f^ ± 0.41
		DN6	0.00195 ^a^ ± 0.00	0.00201 ^b^ ± 0.00
	In	SFA (g/100 g)	41.2 ^a^ ± 0.55	39.7 ^b^ ± 0.61
		PUFA (g/100 g)	12.3 ^e^ ± 0.36	14.5 ^f^ ± 0.52
		UI	0.73 ^c^ ± 0.01	0.77 ^d^ ± 0.01
		N3 (g/100 g)	1.33 ^a^ ± 0.05	1.51 ^b^ ± 0.05
		N6 (g/100 g)	10.9 ^e^ ± 0.31	13.0 ^f^ ± 0.48
		DN6	0.0025 ^e^ ± 0.00	0.0018 ^f^ ± 0.00
VCF		SFA (g/100 g)	48.3 ^a^ ± 0.38	46.4 ^b^ ± 0.81
		PUFA (g/100 g)	9.87 ^e^ ± 0.22	12.2 ^f^ ± 0.52
		UI	0.63 ^c^ ± 0.01	0.68 ^d^ ± 0.01
		N3 (g/100 g)	0.98 ^e^ ± 0.02	1.19 ^f^ ± 0.05
		N6 (g/100 g)	8.89 ^e^ ± 0.20	11.0 ^f^ ± 0.47
		DN6	0.003 ^c^ ± 0.00	0.002 ^d^ ± 0.00
LD	Polar	DN3	1.09 ^a^ ± 0.13	0.81 ^b^ ± 0.05
BF	Neutral	DN6	0.08 ^e^ ± 0.02	0.06 ^f^ ± 0.00
	Polar	MUFA (g/100 g)	20.5 ^c^ ± 0.26	19.8 ^d^ ± 0.14
		UI	1.43 ^c^ ± 0.01	1.46 ^d^ ± 0.01
		MUFA/SFA	0.57 ^c^ ± 0.01	0.55 ^d^ ± 0.00
		N3 (g/100 g)	2.60 ^c^ ± 0.04	2.82 ^d^ ± 0.04
		N6/N3	15.9 ^a^ ± 0.24	14.8 ^b^ ± 0.28
		D9	0.32 ^a^ ± 0.00	0.31 ^b^ ± 0.00
		DN3	0.33 ^a^ ± 0.02	0.38 ^b^ ± 0.01

SCF = subcutaneous fat; LD = *longissimus dorsi*; BF = *biceps femoris*; FA = fatty acids; UI = unsaturation index; MUFA = sum of monounsaturated FA; PUFA = sum of polyunsaturated FA; SFA = sum of saturated fatty acids; DI = desaturation index; D9 = total activity of ∆9 desaturase; DN3 = total activity for the desaturases of n3; DN6 = total activity for the desaturases of n6. Different superscripts indicate significant differences a ≠ b (*p* < 0.05); c ≠ d (*p* < 0.01); e ≠ f (*p* <0.0005).

**Table 5:** Summary of genotype × sex interactions found in body features, metabolic parameters and fatty acid composition when comparing male and female pure Iberian (IB × IB) and Iberian × Large White crossbred pigs (IB × LW).

Age (Days)	Parameter		IB × IB		IB × LW		Gen × Sex
			Females	Males	Females	Males	
0	Body measures	Body weight (kg)	1.27 ± 0.04	1.27 ± 0.05	1.32 ± 0.09	1.52 ± 0.10	+
21	Body measures	Body weight (kg)	4.81 ± 0.15	4.22 ± 0.18	4.51 ± 0.38	4.88 ± 0.34	*
		BPD (cm)	6.03 ± 0.04	5.88 ± 0.06	5.94 ± 0.14	6.19 ± 0.09	**
		ONL (cm)	14.9 ± 0.10	14.8 ± 0.14	13.7 ± 0.34	15.3 ± 1.17	*
		TC (cm)	37.3 ± 0.49	35.7 ± 0.63	35.5 ± 1.17	36.8 ± 1.16	*
		AC (cm)	33.3 ± 0.51	31.1 ± 0.67	30.9 ± 1.11	32.5 ± 0.99	*
60	Body measures	BPD (cm)	7.49 ± 0.09	7.34 ± 0.07	7.81 ± 0.15	8.17 ± 0.13	*
	Viscera weight	Brain (g)	71.7 ± 1.51	71.2 ± 1.39	63.0 ± 2.88	71.7 ± 1.38	+
		Lungs (g)	229 ± 15.4	196 ± 8.87	215 ± 22.3	241 ± 9.32	+
		Pancreas (g)	37.6 ± 3.61	34.3 ± 2.56	36.2 ± 3.87	46.9 ± 2.97	+
		Spleen (g)	54.0 ± 4.39	46.2 ± 3.31	46.6 ± 7.02	58.3 ± 3.35	+
		Kidneys (g)	84.7 ± 5.55	78.5 ± 4.38	74.0 ± 7.86	93.3 ± 3.34	+
	Metabolites	Fruc (mg/dL)	212 ± 14.5	229 ± 9.18	248 ± 14.4	203 ± 3.80	*
	Fatty acids composition	UI BFN	1.09 ± 0.04	1.09 ± 0.03	1.28 ± 0.05	1.05 ± 0.06	+
		N6 BFN (g/100 g)	24.9 ± 1.85	24.8 ± 1.46	31.5 ± 1.26	22.3 ± 2.24	+
		SFA BFN (g/100 g)	39.9 ± 0.19	38.6 ± 0.52	38.8 ± 0.16	39.7 ± 0.38	**
		N6/N3 BFP	9.60 ± 0.27	10.3 ± 0.28	9.28 ± 0.37	9.11 ± 0.42	+
		DN6 LVP (g/100 g)	0.87 ± 0.04	0.75 ± 0.03	0.76 ± 0.04	0.81 ± 0.05	+
210	Metabolites	Fruc (mg/dL)	239 ± 6.87	267 ± 6.12	258 ± 8.69	250 ± 12.2	+
		Urea (mg/dL)	18.5 ± 1.19	18.1 ± 1.92	16.7 ± 2.11	25.9 ± 2.09	**
	Fatty acids composition	MUFA SCFO (g/100 g)	48.9 ± 0.38	47.8 ± 0.41	46.1 ± 0.33	47.7 ± 0.44	**
		PUFA SCFO (g/100 g)	14.1 ± 0.34	13.1 ± 0.44	17.2 ± 0.52	14.7 ± 0.29	+
		MUFA/SFA SCFO	1.32 ± 0.02	1.23 ± 0.02	1.26 ± 0.02	1.27 ± 0.03	+
		N6 SCFO (g/100 g)	12.7 ± 0.31	11.8 ± 0.40	15.5 ± 0.45	13.2 ± 0.27	+
		D9 SCFO	0.56 ± 0.00	0.54 ± 0.00	0.55 ± 0.00	0.55 ± 0.01	+
		DI SCFO	3.61 ± 0.09	3.18 ± 0.10	3.39 ± 0.11	3.53 ± 0.14	*
		MUFA SCFI (g/100 g)	47.2 ± 0.59	45.8 ± 0.43	45.1 ± 0.64	46.2 ± 0.56	*
		MUFA/SFA SCFI	1.20 ± 0.03	1.07 ± 0.02	1.15 ± 0.03	1.16 ± 0.04	+
		N6/N3 SCFI	8.15 ± 0.23	8.48 ± 0.12	9.16 ± 0.12	8.26 ± 0.08	**
		D9 SCFI	0.53 ± 0.01	0.51 ± 0.01	0.53 ± 0.01	0.52 ± 0.01	+
		DI SCFI	2.95 ± 0.09	2.54 ± 0.06	2.90 ± 0.13	2.91 ± 0.14	+
		PUFA VF (g/100 g)	10.3 ± 0.30	9.46 ± 0.26	13.5 ± 0.74	11.1 ± 0.42	+
		N3 VF (g/100 g)	1.01 ± 0.03	0.95 ± 0.02	1.28 ± 0.08	1.10 ± 0.05	+
		N6 VF (g/100 g)	9.27 ± 0.28	8.51 ± 0.25	12.2 ± 0.68	10.0 ± 0.38	+
		PUFA BFN (g/100 g)	7.73 ± 1.77	9.94 ± 1.10	9.47 ± 0.34	8.45 ± 0.41	***
		UI BFN	0.77 ± 0.03	0.82 ± 0.02	0.80 ± 0.01	0.79 ± 0.01	***
		N6 BFN (g/100 g)	6.90 ± 1.69	8.91 ± 1.08	8.50 ± 0.30	7.45 ± 0.41	***
		N6/N3 BFN	8.33 ± 0.75	8.86 ± 0.95	8.84 ± 0.23	7.63 ± 0.61	*
		DN6 BFN	0.06 ± 0.03	0.11 ± 0.02	0.05 ± 0.00	0.06 ± 0.00	***

Gen =genotype; BPD = biparietal diameter; ONL = occipito-nasal length; TC = trunk circumference; AC = abdominal circumference; Fruc=Fructosamine; SFA = sum of saturated fatty acids; MUFA = sum o monounsaturated fatty acids; PUFA = sum of polyunsaturated fatty acids; UI = unsaturation index; D9 = activity of ∆9 desaturase activity; DI = desaturation index; DN3 total activity of the desaturases of n3; DN6 = total activity of the desaturases of n6; BFN = neutral fraction of the *biceps femoris* muscle; BFP = polar fraction of the *biceps femoris* muscle; LVP = polar fraction of the liver; SCFO = outer fraction of the subcutaneous far; SCFI = inner fraction of the subcutaneous fat; VF = visceral fat. + *p* <0.1; * *p* <0.05; ** *p* <0.01; *** *p* <0.001.

## Supplementary Materials

The following are available online at https://www.mdpi.com/2079-7737/9/12/445/s1, Table S1: Calculated analysis (g/kg, dry-matter basis) and fatty acid composition of the piglets diets, Table S2: Body weight (mean kg ± S.E.M.) in pure Iberian (IB × IB) and Iberian and Large White crossbreds (IB × LW) at 0, 21, 60, 90, 120, 150 and 210 days-old, Table S3: Body measures (mean ± S.E.M.) in pure Iberian (IB × IB) and Iberian and Large White crossbreds (IB × LW) at 0, 21, 60 and 210 days-old, Table S4: Organ weights in pure Iberian (IB × IB) and Iberian and Large White crossbreds (IB × LW) at 60 days-old, Table S5: Organ weights in pure Iberian (IB × IB) and Iberian and Large White crossbreds (IB × LW) at 210 days-old, Table S6: Changes over time in mean plasma concentrations (± S.E.M.) for main metabolic parameters in pure Iberian (IB × IB) and Iberian and Large White crossbreds (IB × LW), Table S7: Differences in fatty acid composition (g/100 g ± S.E.M.) and desaturase activity in the outer layer of the subcutaneous fat between pure Iberian (IB × IB) and Iberian and Large White crossbreds (IB × LW) at 60 days-old, Table S8: Differences in fatty acid composition (g/100 g ± S.E.M.) and desaturase activity in the inner layer of the subcutaneous fat between pure Iberian (IB × IB) and Iberian and Large White crossbreds (IB × LW) at 60 days-old, Table S9: Differences in fatty acid composition (g/100 g ± S.E.M.) and desaturase activity in the neutral fraction of the *longissimus dorsi* muscle between pure Iberian (IB × IB) and Iberian and Large White crossbreds (IB × LW) at 60 days-old; Table S10: Differences in fatty acid composition (g/100 g ± S.E.M.) and desaturase activity in the polar fraction of the *longissimus dorsi* muscle between pure Iberian (IB × IB) and Iberian and Large White crossbreds (IB × LW) at 60 days-old, Table S11: Differences in fatty acid composition (g/100 g ± S.E.M.) and desaturase activity in the neutral fraction of the *biceos femoris* muscle between pure Iberian (IB × IB) and Iberian and Large White crossbreds (IB × LW) at 60 days-old, Table S12: Differences in fatty acid composition (g/100 g ± S.E.M.) and desaturase activity in the polar fraction of the *biceos femoris* muscle between pure Iberian (IB × IB) and Iberian and Large White crossbreds (IBxLW) at 60 days-old, Table S13: Differences in fatty acid composition (g/100 g ± S.E.M.) and desaturase activity in the neutral fraction of the liver between pure Iberian (IB × IB) and Iberian and Large White crossbreds (IB × LW) at 60 days-old, Table S14: Differences in fatty acid composition (g/100 g ± S.E.M.) and desaturase activity in the polar fraction of the liver between pure Iberian (IB × IB) and Iberian and Large White crossbreds (IB × LW) at 60 days-old, Table S15: Differences in fatty acid composition (g/100 g ± S.E.M.) and desaturase activity in the outer layer of the subcutaneous fat between pure Iberian (IB × IB) and Iberian and Large White crossbreds (IB × LW) at 210 days-old, Table S16: Differences in fatty acid composition (g/100 g ± S.E.M.) and desaturase activity in the inner layer of the subcutaneous fat between pure Iberian (IB × IB) and Iberian and Large White crossbreds (IB × LW) at 210 days-old, TableS17: Differences in fatty acid composition (g/100 g ± S.E.M.) and desaturase activity in the visceral fat between pure Iberian (IB × IB) and Iberian and Large White crossbreds (IB × LW) at 210 days-old, Table S18: Differences in fatty acid composition (g/100 g ± S.E.M.) and desaturase activity in the neutral fraction of the *longissimus dorsi* muscle between pure Iberian (IB × IB) and Iberian and Large White crossbreds (IB × LW) at 210 days-old, Table S19: Differences in fatty acid composition (g/100 g ± S.E.M.) and desaturase activity in the polar fraction of the *longissimus dorsi* muscle between pure Iberian (IB × IB) and Iberian and Large White crossbreds (IB × LW) at 210 days-old, Table S20: Differences in fatty acid composition (g/100 g ± S.E.M.) and desaturase activity in the neutral fraction of the *biceos femoris* muscle between pure Iberian (IB × IB) and Iberian and Large White crossbreds (IB × LW) at 210 days-old, Table S21: Differences in fatty acid composition (g/100 g ± S.E.M.) and desaturase activity in the polar fraction of the *biceos femoris* muscle between pure Iberian (IB × IB) and Iberian and Large White crossbreds (IB × LW) at 210 days-old, Table S22: Differences in fatty acid composition (g/100 g ± S.E.M.) and desaturase activity in the neutral fraction of the liver between pure Iberian (IB × IB) and Iberian and Large White crossbreds (IB × LW) at 210 days-old, Table S23: Differences in fatty acid composition (g/100 g ± S.E.M.) and desaturase activity in the polar fraction of the liver between pure Iberian (IB × IB) and Iberian and Large White crossbreds (IB × LW) at 210 days-old, Table S24: Differences in fatty acid composition (g/100 g ± S.E.M.) and desaturase activity in the neutral fraction of the liver between pure Iberian (IB × IB) and Iberian and Large White crossbreds (IB × LW) at 210 days-old, Table S25: Differences in fatty acid composition (g/100 g ± S.E.M.) and desaturase activity in the polar fraction of the liver between pure Iberian (IB × IB) and Iberian and Large White crossbreds (IB × LW) at 210 days-old.
